# Modeling heterogeneity in cognitive trajectories in the Framingham Heart Study

**DOI:** 10.3389/fnagi.2025.1471154

**Published:** 2025-06-25

**Authors:** Yuan Fang, Jiachen Chen, Jesse Mez, Claudia L. Satizabal, Michael L. Alosco, Wei Qiao Qiu, Margaret F. Doyle, Joanne M. Murabito, Kathryn L. Lunetta

**Affiliations:** ^1^Department of Pharmaceutical Sciences, Binghamton University School of Pharmacy and Pharmaceutical Sciences, State University of New York, Binghamton, NY, United States; ^2^Department of Biostatistics, Boston University School of Public Health, Boston, MA, United States; ^3^Boston University Alzheimer’s Disease Research Center and CTE Center, Boston University Chobanian and Avedisian School of Medicine, Boston, MA, United States; ^4^Department of Neurology, Boston University Chobanian and Avedisian School of Medicine, Boston, MA, United States; ^5^Framingham Heart Study, National Heart, Lung, and Blood Institute and Boston University Chobanian and Avedisian School of Medicine, Framingham, MA, United States; ^6^Glenn Biggs Institute for Alzheimer’s and Neurodegenerative Diseases, University of Texas Health Science Center at San Antonio, San Antonio, TX, United States; ^7^Department of Anatomy and Neurobiology, Boston University Chobanian and Avedisian School of Medicine, Boston, MA, United States; ^8^Department of Neurology, Boston Medical Center, Boston, MA, United States; ^9^Department of Psychiatry, Boston University Chobanian and Avedisian School of Medicine, Boston, MA, United States; ^10^Department of Pharmacology and Experimental Therapeutics, Boston University Chobanian and Avedisian School of Medicine, Boston, MA, United States; ^11^Department of Pathology and Laboratory Medicine, Larner College of Medicine, University of Vermont, Burlington, VT, United States; ^12^Department of Medicine, Boston University Chobanian and Avedisian School of Medicine, Boston, MA, United States; ^13^Section of General Internal Medicine, Boston Medical Center, Boston, MA, United States

**Keywords:** cognitive decline, cognitive trajectories, Framingham Heart Study (FHS), latent class mixed models (LCMM), clustering

## Abstract

**Introduction:**

The prevalence of cognitive impairment in the population is growing; however, there is substantial heterogeneity in the rate of decline across different cognitive domains. Harmonized factor scores measuring memory, executive function, and language domains have been created in the Framingham Heart Study (FHS).

**Methods:**

This work identified FHS participants with two or more repeated factor scores after age 60 and fitted latent class mixed models (LCMM) to cluster cognitive trajectories within each domain. Non-linear shapes of trajectories were modeled piecewise linearly, followed by stepwise selections to select cluster-specific change points.

**Results:**

We identified different latent classes of participants with early cognitive decline, compared to late decliners, for each domain. Ten-fold cross-validation yielded stable subgroupings. Our findings show latent-class-related differential patterns in cognitive aging in the FHS. We also investigated the association between identified latent classes with existing protein biomarkers of cognitive aging in a subsample of the study and found elevated levels of CD40L and CD14 were associated with a higher risk of early decline in memory and executive function domain, respectively.

**Discussion:**

In summary, our study advances the understanding of cognitive decline heterogeneity among FHS participants and sets the stage for further investigations into early intervention strategies and personalized approaches to mitigate cognitive aging risks.

## 1 Introduction

The number of adults aged 65 and older in the United States is expected to more than double in the next 40 years (Vincent, 2010). This demographic transition raises concerns about a significant rise in cognitive impairment and dementia among this population. Maintaining good cognitive health stands as a pivotal determinant of successful aging, self-sufficiency, and well-being (Gorelick et al., 2017). The objective evaluation of cognitive function involves the utilization of neuropsychological (NP) assessments, facilitating the longitudinal tracking of cognitive performance. Modeling of the longitudinal trajectories of cognitive performance with aging has been well documented in existing literature (Bangen et al., 2019; Hu et al., 2019; Liu et al., 2013; Proust-Lima et al., 2013; Steinerman et al., 2010; van der Willik et al., 2020). Although it is conventionally acknowledged that advanced age generally corresponds to a decline in cognitive function (Harada et al., 2013; Salthouse, 2019), there is a noteworthy degree of heterogeneity in the rate of cognitive decline observed within cohorts of older adults of the same age (Wilson et al., 2002). Substantial heterogeneity in the pace of cognitive decline across different cognitive domains has been observed as well (Goh et al., 2012; Lövdén et al., 2005; Small et al., 2011). Variability in cognitive decline within and across different cognitive domains has been related to different likelihoods of progression to Alzheimer’s disease (AD) (Cloutier et al., 2015). While numerous statistical/machine learning techniques have been utilized to discover risk stratification or disease progression subgroups based on participants’ demographic and biomarker profiles and subsequently offering *post-hoc* summaries of cognitive trajectories within each subgroup (Dong et al., 2017; Racine et al., 2016; Young et al., 2018), limited efforts have been directed toward directly clustering the trajectories of cognitive functions.

The inherent patterns of change in specific cognitive domains might suggest potential subtypes of AD and other dementias. The identification of latent classes in cognitive trajectories with aging thus may provide insights into early disease course modification before dementia onset or prior to clinical symptoms (Howlett et al., 2021). In addition, by clustering the cognitive trajectories directly, individuals can be placed into more homogenous subgroups, which may also facilitate the recognition of latent groups with an elevated risk of progressing to AD dementia. Such latent subgroupings could serve as a preclinical substitute for cognitive outcomes or offer valuable insights for participant categorization in AD prevention trials (Ritchie et al., 2016; Watts, 2018). Recently, latent class analysis has been directly applied to cognitive trajectories, revealing distinct subgroups of participants, each characterized by a unique pattern of cognitive function changes (Geifman et al., 2018; Howlett et al., 2021; Sebastiani et al., 2020; Villeneuve et al., 2019). While some prior studies have employed extensive neuropsychological batteries and constructed domain-level composite or factor scores (Geifman et al., 2018; Sebastiani et al., 2020), many have still focused on one or two cognitive domains, included smaller sizes, or relied on just two measurements per individual, which could oversimplify the modeling of cognitive trajectories by assuming a linear cognitive decline with age.

Identifying data sets with comprehensive longitudinal cognitive function assessment and choosing appropriate statistical analyses have represented the two major challenges in the pattern discovery of cognitive decline (Sebastiani et al., 2020). Various longitudinal cohorts have utilized distinct NP test batteries and protocols, which makes the combination of NP performance data across cohorts non-trivial. The Framingham Heart Study (FHS) is a large community-based study with multiple sub-cohorts, which has comprehensively phenotyped cognitive outcomes through repeated NP assessments conducted by trained examiners under strict quality control (Au et al., 2010). Recently, separate harmonized factor scores that measure memory, executive function and language domains have been established using structural equation modeling (Scollard et al., 2023). These scores facilitate the integration of longitudinal cognitive profiles across multiple FHS cohorts that had a growing test battery over time.

In this study, we aim to leverage the comprehensive set of NP tests administered to the FHS cohorts and implement the newly developed harmonized cognitive factor scores for memory, executive function, and language domains. Here we describe an innovative, flexible method for modeling longitudinal cognitive profiles within the FHS. It accommodates group-specific and domain-specific changes in cognitive decline rates at different ages, while clustering participants into latent subgroups based on their cognitive factor score trajectories. We characterize participants in each identified subgroup based on their distinct demographic profiles and examine the correlation between the identified subgroups and existing aging biomarkers, aiming to provide a more accurate and comprehensive approach to assessing cognitive aging.

## 2 Materials and methods

### 2.1 Study sample

The FHS is a community-based prospective cohort study, which recruited 5,209 participants as the Original cohort (Gen 1) in 1948 to investigate the risk of cardiovascular disease (CVD) (Dawber and Kannel, 1966). The Offspring cohort (Gen 2), recruited in 1971, consists of 5,124 participants who have at least one parent in the Original cohort and some of their spouses (Feinleib et al., 1975; Kannel et al., 1979). In 1994 the FHS further recruited 506 multi-ethnic participants as the Omni 1 cohort to reflect the greater racial and ethnic diversity in the town of Framingham (Tsao and Vasan, 2015). All cohorts have received routine examinations for CVD and related risk factors. The Gen 1 participants were invited to take a battery of neuropsychological (NP) tests between examinations 14 and 15 (1976–1978) (Elias et al., 1997). The initial NP battery administered to Gen 1 participants was at a core visit, while starting in 1981, NP batteries were administered during ancillary exams (Elias et al., 1997). The Gen 2 and Omni 1 cohorts were also invited to undergo NP testing starting 1999 and 2000, between their regular examinations 7–8 and 2–3, respectively, and repeatedly around every 5 years during ancillary exams (Au et al., 2010). However, the protocol until 1999 was to only administer the NP battery to those flagged as potentially cognitively impaired (Scollard et al., 2023), therefore, the Gen 1 participants who experienced cognitive decline were expected to have more frequent NP tests. All FHS participants were invited to take regular follow-up NP tests to help detect changes in their cognitive functioning but not all chose to attend the additional visits (Au et al., 2010; Satizabal et al., 2016; Scollard et al., 2023).

**FIGURE 1:**
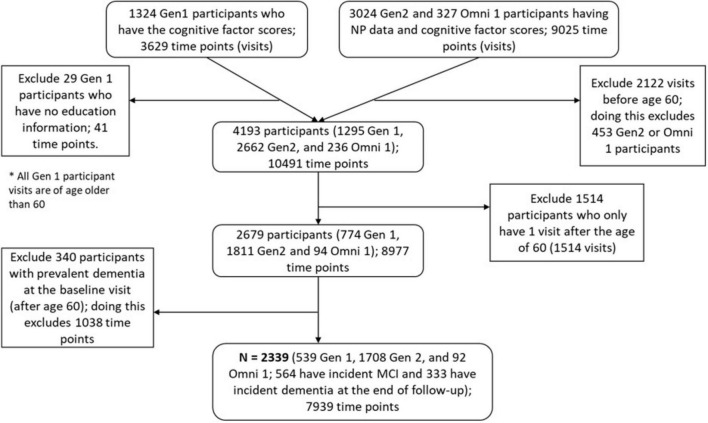
Sample selection flowchart.

In this study, we included 2,339 FHS participants, 539 from Gen 1, 1,708 from Gen 2, and 92 from Omni 1, to investigate subgroups of differential patterns in the longitudinal cognitive function changes. We included data obtained after the age of 60 years from each participant and excluded participants with only one NP test visit after age 60, with a prevalent dementia diagnosis at their baseline visit for this study (first visit at or after age 60), or those without education data. The resulting total number of time points to be studied is 7,939 from 2,339 participants. Details of sample selection can be found in the flow chart in Figure 1. All participants provided written informed consent at each attended examination and at each NP testing; FHS exams are reviewed by the Institutional Review Board at Boston University Medical Center.

### 2.2 Overview of the neuropsychological tests and domain factor scores in the FHS

All participants from the FHS cohorts were invited to undergo a comprehensive battery of NP tests although not all chose to attend. The NP tests were carried out by trained psychometricians following standard administration protocols. This initiative was undertaken as part of a larger study with the primary objective of establishing foundational measures of brain structure and cognition (Au et al., 2010). Detailed information concerning the NP test batteries employed in the FHS cohorts could be found in the existing literature (Au et al., 2010; Elias et al., 1997; Satizabal et al., 2016; Scollard et al., 2023).

Broadly, NP tests included in the battery are representative of the following four different cognitive domains: verbal and visual episodic memory, attention and executive function, language, and visuospatial abilities. Monitoring NP test performance provided a way of measuring and monitoring cognitive changes. However, different cohorts in the FHS went through different waves of NP test batteries. The NP battery in the FHS has grown over time. The initial battery administered to the Gen 1 cohort included fewer tests than the batteries the Gen 2, Omni 1 cohorts underwent, leading to difficulties in combining or comparing across the FHS cohorts (Scollard et al., 2023). To address this problem, calibrated and harmonized factor scores for the memory, executive function, and language domains were developed based on NP test batteries and items from Mini-Mental State Examination (MMSE) or Consortium to Establish a Registry for Alzheimer’s Disease, using data across all FHS NP test visits (Scollard et al., 2023). The NP tests and MMSE components included in developing each harmonized domain factor score for the memory, executive function, and language domains can be found in the developing paper (Scollard et al., 2023). Due to insufficient NP tests in the visuospatial domain, no harmonized factor score was composed for this domain. These calibrated and harmonized domain factor scores (referred to as the “factor scores” hereafter), quantify cognitive performance on the same scale across the cohorts, and minimize ceiling effects observed in raw NP test performance, facilitating pooled analyses across the FHS cohorts (Scollard et al., 2023).

### 2.3 Statistical analysis

#### 2.3.1 The piecewise linear latent-class mixed effect models

For each of the three cognitive domains (memory, executive function, and language) with available longitudinal factor scores, we identified distinct trajectory classes using piecewise linear latent-class mixed effect models (LCMMs). The linear mixed-effect models (LMM) (Laird and Ware, 1982) is a standard statistical procedure to model trajectories of changes while allowing for both within-subject and between-subject variation. The LCMM is a finite mixture of LMMs, where the responses are assumed to be from a mixture of Gaussian distributions with group means described by LMM regression functions (Muthén and Shedden, 1999; Proust and Jacqmin-Gadda, 2005). As a model-based clustering framework, an LCMM can classify participants based on the shape of the trajectories of interest, and has been recently applied to model the heterogeneities in cognitive decline, dementia, and preclinical Alzheimer’s Diseases in several longitudinal cohorts (Geifman et al., 2018; Gonzales et al., 2020; Howlett et al., 2021; Proust-Lima et al., 2016; Villeneuve et al., 2019) and to study differential progression patterns in other diseases (Fang et al., 2023).

Cognitive decline is a complex process and typically shows a non-linear trend along age or time in the study, hence only fitting linear effects with age or time would over-simplify the model. While LCMMs are a flexible tool for identifying heterogeneous longitudinal patterns, caution is necessary when the trajectories are non-linear. One common practice is to assume a polynomial function of time (Fang et al., 2023; Proust-Lima et al., 2016). However, the polynomial-time-function term may also force the mean trajectory profile shapes to not make practical sense. Additionally, LCMMs typically suffers from computational convergence issues, especially when the number of model parameters to be estimated is large. To address these two issues, we proposed a piecewise linear LCMM to allow for group-specific change points and assume a linear change in outcome over the time segments with different slopes before and after the change points. We used a small, predefined change points sets to reduce the number of parameters to estimate and avoid the computational convergence issue. These age thresholds were selected based on clinical knowledge and prior literature suggesting inflection points in cognitive aging and preclinical Alzheimer’s disease progression (Salthouse, 2019; Sperling et al., 2011).

#### 2.3.2 Model-building and selection

For each of the three cognitive domains, we followed the same procedure to build a piecewise linear LCMM. Mathematical details of the construction of the model can be found in Supplementary material section S1. Details for model specification of the piecewise linear LCMMs can be found in Supplementary material section S2. Briefly, we built models for the longitudinal factor scores with two sets of predefined change points, one at ages of 65, 75, and 85 years and another at ages of 70, 80, and 90 years. We included group-specific fixed effects for age, sex, and education level in all models. The age terms were broken down to piecewise linear with changes of slopes before and after the prespecified change points. We included individual random effects to allow individual trajectories to vary from the group-specific mean. We fit models for G = 1–4 latent groups, yielding a total of 8 models (G groups times change points at the two sets of ages). Models with more than 4 groups often failed to converge or yielded small subgroups with insufficient samples for downstream characterization and comparison and unstable parameter estimates, which limited their interpretability. For each of the 8 complete models, we used a backward selection algorithm, with details described in Supplementary material section S3, to remove unneeded change points.

LCMMs are finite mixtures of regression models, and the true number of components G is not known. We used the Bayesian Information Criterion (BIC) (Schwarz, 1978) to select the final best model from among the 8 models. The flexibility introduced by combining group-specific fixed effects and individual random effects with the backward selection method ultimately allows for the selection of group-specific change points for different cognitive domains.

#### 2.3.3 Sensitivity analysis: 10-fold cross-validation for model robustness

To evaluate the robustness of the selected change points, the piecewise linear structure, and the identified number of groups, we performed a sensitivity analysis using 10-fold cross-validation for each of the three cognitive function domains. We randomly partitioned the study participants into 10 subsamples. We used 9 portions as the training data to fit a piecewise linear LCMM described above, obtained the model with the best BIC; then, the left over 1 portion was treated as the testing data and the best model identified in the training data were applied to predict the latent classes. This training-testing split was repeated for 10 iterations until each subsample was treated as the testing data once. After the 10 training-testing model fittings and class predictions, the number of groups selected for the training data sets, as well as the median predicted soft classifications (posterior probability of belonging to the predicted classes) were summarized to determine whether the models selected by BIC were stable. We summarized the average adjusted Rand Index (ARI) (Hubert and Arabie, 1985) across the 10 testing sets comparing class membership predicted by the training model and the original model fitted using the whole dataset to determine on the robustness of the model fitted.

#### 2.3.4 Association with existing plasma biomarkers

To validate that the latent classes identified using the proposed piecewise linear LCMM can be applied as an alternative cognitive outcome, we further investigated the association between the identified latent classes and previously measured plasma protein inflammatory biomarkers in a subsample of the Offspring cohort. At the Offspring cohort routine exam seven, 85 plasma proteins were measured using the Systems Approach to Biomarker Research (Ho et al., 2018). A previous study from our group established associations of several inflammatory protein biomarkers from these 85 with cognitive test performance, brain MRI volumes, and incident dementia (Fang et al., 2022). Here, we also examine the association between the inflammatory protein biomarkers and the identified latent classes in cognitive factor scores across the three domains using logistic regression models. We identified a sample of 1,617 Offspring cohort participants who attended the seventh routine examination, where the protein biomarkers were profiled. For each cognitive domain, the latent classes were grouped into a dichotomous response variable representing early fast decline (coded as 1) vs. steady or late fast decline (coded as 0). The protein biomarker levels were normalized to mean 0 and standard deviation 1. We employed a logistic regression model, adjusted for age at the cognitive test visit closest to exam 7 after age 60, the time difference between this visit and exam 7 in years, sex, education level, and *APOE* ϵ4 carrier status. A robust standard error estimate was used to account for familial correlation. We tested 5 protein biomarkers that showed significant association with cross-sectional cognitive scores in our previous study (Fang et al., 2022): monocyte differentiation antigen (CD14), CD5 molecule-like (CD5L), soluble CD40 ligand (CD40L), soluble receptor for advanced glycation end products (sRAGE), and myeloperoxidase (MPO). Additionally, a sensitivity analysis was performed on a subsample of 906 participants, whose cognitive test closest to exam 7 after age 60 was within 2 years after exam 7, using the same model and covariates.

#### 2.3.5 Analysis software

All analyses were carried out using the statistical software R version 4.0.2. The piecewise linear LCMMs were fitted with the hlme function from package lcmm version 1.9.3. The backward selection algorithm was written and implemented in R. ARIs were calculated using the adjustedRandIndex function from the mclust package version 5.4.10. Associations between protein biomarkers and the latent classes were fitted using the glm function from the stats package together with the sandwich package version 3.1-0 and the lmtest package version 0.9-40 for the robust standard error estimation. All figures from the model were created using ggplot2 package version 3.3.6.

## 3 Results

### 3.1 Latent classes identified from clustering the trajectories of three cognitive domain factor scores

#### 3.1.1 Latent classes identified from the memory factor score trajectories

**FIGURE 2:**
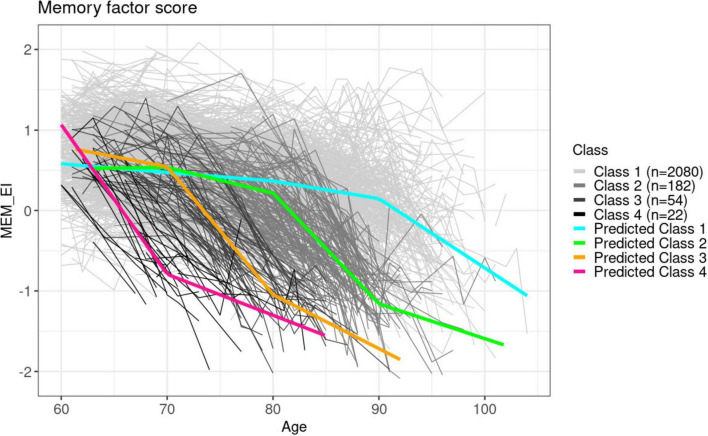
Predicted mean trajectories superposed on observed trajectories for each latent subgroup identified in the memory domain. The gray shaded trajectories are the true observations, whereas the colored lines are the mean predicted trajectories from each subclass.

Overall participant demographics are summarized in Table 1. Our piecewise linear LCMM model identified 4 latent subgroups based on the memory domain factor score trajectories. Figure 2 illustrates the predicted mean profile for each subgroup superposed on the actual trajectory of individuals within the corresponding subgroup. Each of the four identified latent subgroups exhibited distinct patterns, characterized by changes in rates of decline in the memory domain factor scores around the age of 70 (class 3, early decline group), 80 (class 2 late decline group), and 90 (class 1, latest decline group) years. In contrast, participants in class 4 (earliest decline group) demonstrated a steep decline starting around the age of 60, which is the baseline of the study.

**TABLE 1:** Participant demographics for all participants.

Demographic		All participants (*N* = 2,339)
Basic demographics	Female, *n* (%)	1,321 (56.5)
	*APOE* ϵ4 carrier, *n* (%)	388 (16.6)
	Attended college, *n* (%)	1,487 (63.6)
	Number of NP visits, median (IQR)	3 (2)
	Median years apart between tests, mean (SD)	5 (4)
	Have NP tests after dementia, *n* (%)	247 (10.6)
Baseline characteristics	Age, mean (SD)	69 (8)
	MMSE, median (IQR)	29 (2)
	BMI, mean (SD)	28 (5)
	SBP, mean (SD)	132 (19)
	DBP, mean (SD)	73 (10)
	Hypertension Rx, *n* (%)	1,006 (43.0)
	Total cholesterol, mean (SD)	196 (36)
	Blood glucose, mean (SD)	105 (30)
	Diabetes Rx, *n* (%)	172 (7.4)
	Smoking, *n* (%)	183 (77.8)
Incident cognitive outcomes	Incident MCI, *n* (%)	597 (25.5)
	Incident MCI but not dementia, *n* (%)	225 (9.6)
	Incident dementia, *n* (%)	372 (15.9)
Survival information	Age at last exam visit, mean (SD)	79 (8)
	Years between last exam visit and last contact, mean (SD)	4 (4)
	Alive till the end of 2019, *n* (%)	1,347 (57.6)

All incident cognitive events were counted from baseline to the end of 2019. *APOE*, Apolipoprotein E; NP, Neuropsychological; IQR, Inter quartile range; SD, Standard deviation; MMSE, Mini-Mental State Examination; BMI, Body mass index; SBP, Systolic blood pressure; DBP, Diastolic blood pressure; MCI, Mild cognitive impairment.

Table 2 provides a summary of the participant demographics for each identified latent class. Average baseline ages are similar for classes 1 and 3 (age 69 and 68, respectively), whereas the average age at the first exam was earlier for the earliest decline group class 4 (age 64) and later for class 2 (75 years). Cognitive status as measured by the MMSE was similar among all participants at the baseline exam. However, the proportions of *APOE* ϵ4 carriers, and of incident dementia and MCI in the two early decline groups (classes 3 and 4) were greater over the entire follow up period compared to the later decline groups. Participants from class 4, the earliest decline latent group identified, also shows on average the shortest time in years between each NP tests during the full follow-up, which is expected due to the protocol that participants experiencing cognitive impairment were tested more frequently.

**TABLE 2:** Demographic for subgroups identified in all three domains.

		Memory domain predicted latent classes				Executive function domain predicted latent classes		Language domain predicted latent classes		
Demographic		Class 1 (*n* = 2,080)	Class 2 (*n* = 182)	Class 3 (*n* = 54)	Class 4 (*n* = 22)	Class 1 (*n* = 2,302)	Class 2 (*n* = 36)	Class 1 (*n* = 2,196)	Class 2 (*n* = 92)	Class 3 (*n* = 50)
Basic demographics	Female, *n* (%)	1,154 (56)	119 (65)	34 (63)	13 (59)	1,302 (57)	18 (50)	1,254 (57)	39 (42)	27 (54)
	*APOE* ϵ4 carrier, *n* (%)	338 (16)	18 (10)	20 (37)	12 (55)	374 (16)	14 (39)	364 (17)	13 (14)	11 (22)
	Attended college, *n* (%)	1,335 (64)	98 (54)	37 (69)	16 (73)	1,455 (63)	31 (86)	1,388 (63)	71 (77)	27 (54)
	Number of NP visits, median (IQR)	3 (2)	4 (2.8)	4 (2)	3 (2)	3 (2)	3 (2)	3 (2)	3 (3)	4 (2)
	Median years apart between tests, mean (SD)	5 (4)	3.5 (4)	3.8 (4)	3.2 (2.5)	5 (4)	5 (3.6)	5 (4)	5 (5)	3 (4.4)
	Have NP tests after dementia, *n* (%)	99 (5)	100 (55)	35 (65)	13 (59)	225 (10)	22 (61)	194 (9)	22 (24)	31 (62)
	Age, mean (SD)	69 (8)	75 (6)	68 (5)	64 (3)	69 (8)	66 (4)	69 (8)	71 (8)	69 (7)
	MMSE, median (IQR)	29 (2)	29 (2)	29 (2)	29 (1)	29 (2)	29 (2)	29 (2)	29 (1.5)	29.5 (1.8)
	BMI, mean (SD)	28 (5)	27 (5)	27 (5)	29 (5)	28 (5)	28 (5)	28 (5)	27 (4)	27 (5)
	SBP, mean (SD)	131 (19)	138 (21)	130 (20)	128 (18)	132 (19)	135 (21)	132 (19)	133 (19)	135 (24)
	DBP, mean (SD)	73 (10)	73 (10)	73 (9)	74 (10)	73 (10)	74 (10)	73 (10)	74 (11)	74 (10)
	Hypertension Rx, *n* (%)	900 (43)	74 (41)	23 (43)	9 (41)	989 (43)	17 (47)	949 (43)	38 (41)	19 (38)
	Total cholesterol, mean (SD)	196 (36)	203 (40)	187 (28)	193 (38)	196 (36)	199 (35)	196 (36)	195 (41)	199 (32)
	Blood glucose, mean (SD)	105 (29)	105 (37)	104 (29)	110 (27)	105 (30)	107 (29)	105 (29)	99 (21)	120 (62)
	Diabetes Rx, *n* (%)	157 (8)	8 (4)	4 (7)	3 (14)	166 (7)	6 (17)	159 (7)	7 (8)	6 (12)
	Smoking, *n* (%)	159 (8)	13 (7)	8 (15)	3 (14)	178 (8)	5 (14)	173 (8)	7 (8)	3 (6)
Incident cognitive outcomes	Incident MCI, *n* (%)	399 (20)	132 (73)	47 (87)	19 (87)	568 (25)	29 (81)	521 (24)	39 (42)	37 (74)
	Incident MCI but not dementia, *n* (%)	197 (10)	16 (9)	7 (13)	5 (23)	220 (10)	5 (14)	212 (10)	11 (12)	2 (4)
	Incident dementia, *n* (%)	202 (10)	116 (64)	40 (74)	14 (64)	348 (15)	24 (67)	309 (14)	28 (30)	35 (70)
Survival information	Age at last exam visit, mean (SD)	79 (8)	87 (4)	80 (4)	73 (4)	79 (8)	76 (5)	79 (8)	82 (9)	80 (7)
	Years between last exam visit and last contact, mean (SD)	4 (4)	2 (3)	2 (2)	4 (3)	4 (4)	3 (3)	4 (4)	4 (3)	2 (2)
	Alive till the end of 2019, *n* (%)	1,267 (61)	50 (28)	22 (41)	8 (36)	1,334 (58)	13 (36)	1,290 (59)	43 (47)	14 (28)

All incident cognitive events were counted from baseline to the end of 2019. *APOE*, Apolipoprotein E; NP, Neuropsychological; IQR, Inter quartile range; SD, Standard deviation; MMSE, Mini-Mental State Examination; BMI, Body mass index; SBP, Systolic blood pressure; DBP, Diastolic blood pressure; MCI, Mild cognitive impairment.

Among the two later decline groups (classes 1 and 2), class 2 identified participants with generally older age at baseline, paired with a higher proportion of participants experiencing dementia or MCI onset during follow-up. The mean predicted decline rate for class 2 is similar to those observed for class 3 but occurred at a later age. The late decline group class 2 contains participants with the oldest baseline age and the lowest proportion of participants attended college (54%), which is an indication that it grouped more Original cohort participants. On the other hand, class 1, the largest group, included participants whose memory domain cognitive function remained relatively stable until the age of 90. This group is also characterized by the lowest proportion of *APOE* ϵ4 carriers, dementia or MCI onset, and the longest time between each NP visit.

#### 3.1.2 Latent classes identified from the executive function factor score trajectories

**FIGURE 3:**
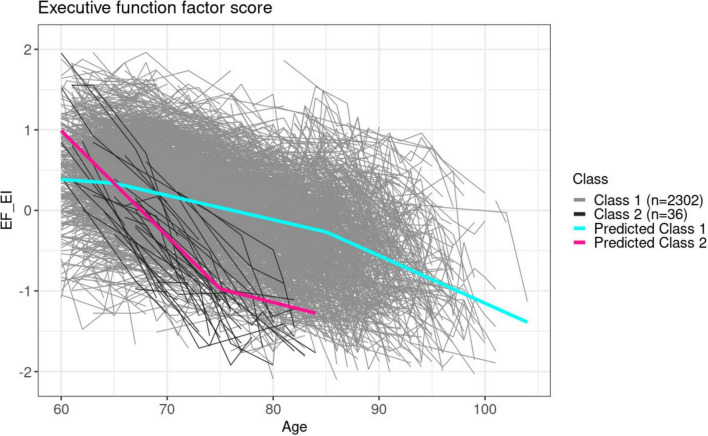
Predicted mean trajectories superposed on observed trajectories for each latent subgroup identified in the executive function domain. The gray shaded trajectories are the true observations, whereas the colored lines are the mean predicted trajectories from each subclass.

Our modeling identified two latent classes based on the factor score trajectories from the executive function domain. Predicted mean trajectories for each latent subgroup identified overlaid on the observed actual individual trajectories in the executive function domain are visualized in Figure 3. Participant demographics for each latent subgroup are summarized in Table 2.

Class 1 (*n* = 2,302) was characterized by a relatively consistent decline in overall executive function performance, marked by subtle alterations in the rate of decline around ages of 65 and 85 years. In contrast, the much smaller class 2 (*n* = 36) had a rapid decline from the baseline age of 60 years. The average age of the earlier, rapid decline class 2 at baseline was 3 years younger than the later, slower decline class 1 group. The two classes had similar baseline MMSE scores, and other baseline characteristics of the participants between these two groups are also similar. However, the earlier, faster decline group (class 2) had a higher proportion of *APOE* ϵ4 carriers and greater proportion of incidence of dementia and MCI during follow-up. Additionally, participants identified within the early decline latent subgroup experienced more deaths during follow up, with 36% surviving to the end of 2019 (end of the follow up), compared to 58% of the slower decline group. Similar to observed in the memory domain, the group with older baseline age also included a lower overall education level.

#### 3.1.3 Latent classes identified from the language factor score trajectories

**FIGURE 4:**
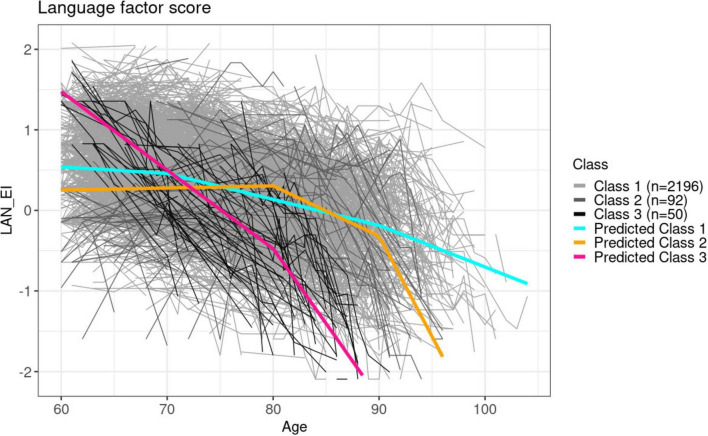
Predicted mean trajectories superposed on observed trajectories for each latent subgroup identified in the language domain. The gray shaded trajectories are the true observations, whereas the colored lines are the mean predicted trajectories from each subclass.

Our modeling identified three latent subgroups when clustering based on the language domain factor score trajectories. Figure 4 visually represents the predicted mean trajectories and observed trajectories for each identified latent subgroup in the language domain, while participant demographics for each subgroup are summarized in Table 2.

Participants identified within the latent class 1 (*n* = 2,196) exhibit a consistent decline throughout the entire follow-up with small increases in decline rate around the ages of 70 and 90. Class 3 (*n* = 50) exhibited an early steep decline starting at the beginning of the observed age range (age 60 years) followed by a steeper decline after age 80 years. The participants in class 2 (*n* = 92) had no decline until age 80, and then an increased decline rate after age 90 years.

Baseline characteristics of participants in the three latent groups differed. The late decline group (class 2) had the lowest proportion of *APOE* ϵ4 carriers (14%) and the early decline group (class 3) had the highest proportion (22%), but these differences between classes were smaller than for the memory and executive function domains. Consistent with findings in other two domains, the early decline group has the highest proportion of participants who developed dementia or MCI during follow-up. However, the late decline group (class 2) had a higher rate of incident MCI and dementia than the consistent decline group. Different from observations from the memory and executive function domain, where higher proportions of attended college appears more in groups with younger baseline age, the late fast decline group in the language domain identified higher proportions of participants who attend college but with the oldest baseline age as compared to the other groups.

#### 3.1.4 10-fold cross-validation

We employed a 10-fold cross-validation approach to assess the robustness of the identified subgroups within each cognitive domain. Results of the 10-fold cross-validation for the memory, executive function, and language domains are detailed in Supplementary Tables S1–S3, separately.

In the memory domain, 6 out of 10 rounds of fitting the training data resulted in a 4-group solution, consistent with the findings from fitting the entire dataset. In these 6 rounds, when applying the model obtained from the training data to the testing data, the predicted cluster membership closely aligned with the whole-data model, with ARIs close to 1. In the remaining 4 rounds, where fitting the training data did not select a 4-component model, the best model favored 3 subgroups. Across all 10 rounds, the median posterior probability for all observations exceeded 0.74, indicating a robust and definite classification.

In the executive function domain, the 2-component piecewise linear LCMM model identified when fitting the entire dataset demonstrated high robustness. Among the 10 cross-validation rounds, 9 yielded a 2-component model when fitting the training data. In 8 out of these 9 rounds, the predicted class memberships showed ARIs close to 1 when comparing the class labels obtained by applying training data model to the testing data and the class labels predicted from the whole-data model. The median posterior probabilities were consistently close to 1 across all rounds, suggesting a definitive classification. The single round where fitting training data did not select a 2-component model did not prefer other models either; however, computational issues prevent the convergence even with a one-component piecewise linear LMM.

In the language domain, the latent classes identified when fitting the full dataset also proved to be robust. All 10 cross-validation rounds result in 3-component models when fitting the training data. Among these rounds, 8 models obtained from the training data predict the testing data class labels similar to the full-data model with ARIs above 0.86 and an average close to 0.9. In addition, the median posterior probabilities are also close to 0.9, indicating high confidence in the classification. For the remaining 2 rounds, although the best model selected when fitting the training data are 3-group solutions, they differed from the 3-group model obtained when fitting the entire dataset. The ARI between classifications on the testing data are only around 0.3, indicating poor alignment and the median posterior probabilities also drop from around 0.9 to approximately 0.7.

#### 3.1.5 Comparison of latent classes across cognitive domains

For each of the three cognitive domains we investigated, the proposed piecewise linear functions identified two overarching patterns of cognitive decline among the FHS participants. For each cognitive domain, one or two latent subgroups were characterized by an early rapid decline (class 2 and 3 in the memory domain, class 2 in the executive function domain, and class 3 in the language domain), while the other classes exhibited either steady decline or late-onset rapid decline (classes 1 and 4 in memory, class 1 in executive function, and classes 1 and 2 in language).

Table 3 presents crosstabulations of the identified latent subgroups across three cognitive domains. In all three comparisons, the majority of the late decline groups comprised the same participants across the domains. In contrast, the early decliners in each domain are not consistently the same participants. Specifically, about 61% of the early decliners in executive function domain also exhibited early signs of fast decline in their memory domain, and 44% of the early decliners in language cognitive function also similarly showed early signs of decline in their memory domain. However, only 10 participants demonstrated early decline in both executive function (28%) and language (20%).

**TABLE 3:** Predicted class membership across memory, executive function, and language domains: a) memory domain vs. executive function domain; b) memory domain vs. language domain; c) executive function domain vs. language domain.

(a)		Executive function domain predicted latent classes		
		Class 1		Class 2
**Memory domain predicted latent classes**	Class 1	2,068		12
	Class 2	180		2
	Class 3	43		11
	Class 4	11		11
**(b)**		Language domain predicted latent classes		
		Class 1	Class 2	Class 3
**Memory domain predicted latent classes**	Class 1	1,988	69	13
	Class 2	150	17	15
	Class 3	35	2	17
	Class 4	13	4	5
**(c)**		Language domain predicted latent classes		
**Executive function domain predicted latent classes**		Class 1	Class 2	Class 3
	Class 1	2,176	86	40
	Class 2	20	6	10

### 3.2 Association of clinical outcomes with the latent classes

#### 3.2.1 Association of early vs. late decline with incident MCI and dementia

Table 4 summarizes the distribution of individuals across early decline groups (0, 1, 2, or all 3 domains) and compares this with the clinical diagnoses of MCI and dementia during follow up through 2019. A total of 2,221 participants (about 95% from the study sample) were not clustered into the early decline group for any domain. Of these participants, 77% did not develop MCI or dementia. Among the 81 participants who were identified as early decliners for one cognitive domain, 70% developed either MCI (19%) or dementia (51%) during follow-up. For the 27 participants clustered into early decline groups for 2 domains, 82% developed dementia, and 7% developed MCI without dementia. Only 9 participants across all domains were clustered into early decline groups for all three domains, and all these individuals were diagnosed with dementia during follow up.

**TABLE 4:** The number of domains for which an individual was clustered into an early decline group vs. clinical diagnosis of MCI or dementia.

		No MCI or dementia onset (%)	Incident MCI but no dementia (%)	Incident dementia (%)
**Number of domains an individual got clustered into the early decline latent classes**	0	1,714 (77)	208 (10)	299 (13)
	1	24 (30)	15 (19)	42 (51)
	2	3 (11)	2 (7)	22 (82)
	3	0 (0)	0 (0)	9 (100)

Early decline latent classes are groups featuring faster decline prior to age of 75 years. These latent classes include: Memory domain class 2 and class 3, executive function domain class 2, and language domain class 3.

#### 3.2.2 Association with existing plasma inflammatory protein biomarkers

In the Offspring cohort subsample of this study, we investigated the association between a set of 5 protein biomarkers that were significantly associated with NP performance in our previous study (Ho et al., 2018) and the dichotomized latent classes (early rapid decline vs. late or steady decline) for each of the three cognitive domains adjusting for age, sex, education level, and *APOE* ϵ4 carrier status. Tables 5, 6 summarize the estimated odds ratios, standard errors, and *p*-values for the associations between the 5 protein biomarkers and the subclass membership for the memory domain and the executive function domain, respectively. Results for the language domain are summarized in Supplementary Table S4.

**TABLE 5:** Association of the protein biomarkers with the piecewise linear LCMM identified subclasses as early decline vs. late decline in the memory domain.

Biomarker	Odds ratio	Standard error	*p*-value
Main analysis sample (*n* = 1,617)			
CD14	0.97	0.16	0.84
CD5L	0.96	0.11	0.72
CD40L	1.42	0.16	0.03
sRAGE	0.85	0.13	0.19
MPO	0.99	0.14	0.97
Sensitivity analysis sample (*n* = 907)			
CD14	1.19	0.19	0.39
CD5L	0.94	0.13	0.66
CD40L	1.40	0.20	0.08
sRAGE	0.82	0.15	0.18
MPO	0.95	0.17	0.78

**TABLE 6:** Association of the protein biomarkers with the piecewise linear LCMM identified subclasses as early decline vs. late decline in the executive function domain.

Biomarker	Odds ratio	Standard error	*p*-value
Main analysis sample (*n* = 1,617)			
CD14	1.38	0.18	0.08
CD5L	0.87	0.12	0.27
CD40L	0.68	0.22	0.09
sRAGE	0.86	0.20	0.44
MPO	0.93	0.12	0.57
Sensitivity analysis sample (*n* = 907)			
CD14	1.57	0.22	0.04
CD5L	0.89	0.15	0.44
CD40L	0.56	0.30	0.06
sRAGE	0.82	0.25	0.41
MPO	0.95	0.14	0.71

For the memory domain, higher CD40L was nominally associated with higher odds of being clustered into the early decline group (estimated odds ratio 1.42). This trend persisted but was not significant in the smaller sensitivity analyses sample with ages closer to those at exam 7. For the executive function domain, while no signal was observed from the main analysis sample, higher CD14 exhibited a nominally significant positive association in the subsample closer in age to exam 7, indicating higher odds of being clustered into the early decline group as compared to the late decline group (estimated odds ratio as 1.57). In addition, although not significant, higher CD40L showed a trend of association with lower odds (estimated odds ratio 0.56, *p*-value = 0.06) of being classified as early decliners in the executive function domain. No other significant associations were identified except for CD14 and CD40L.

## 4 Discussion

In this study, we described a flexible way of modeling the heterogeneity in cognitive decline among FHS participants from multiple cohorts. By utilizing the newly developed cognitive domain factor scores in the FHS, we combined participants across different FHS cohorts, ensuring a large sample size. The proposed method leverages the LCMM to cluster participants into more homogeneous groups based on their longitudinal profiles of cognitive factor scores. The piecewise linear component, together with change point selection, provides flexibility to account for group-specific and domain-specific non-linearity in cognitive decline. The identified subgroups are stable and robust, illustrated by 10-fold cross-validation.

Each identified subgroup exhibited distinct patterns of cognitive decline across memory, executive function, and language domains, characterized by unique participant demographics, including baseline age, education level, and *APOE* ϵ4 carrier status. Specifically, higher education level is enriched in the subgroup of participants with a late decline in their language domain cognitive functions. This aligns with the existing findings that education has positive effects on cognitive function and individuals with higher educational attainment are likely to experience less cognitive decline as they age (Lövdén et al., 2020; Zahodne et al., 2015).

While most literature studying heterogeneity in cognitive aging discovered subgroups with different rates of cognitive function decline (Howlett et al., 2021), ages of dementia or AD onset (Villeneuve et al., 2019), or studies of which cognitive domains are disproportionately affected, it is challenging to bring all aspect together. Our study is distinguished from the literature by its identification of subgroups with different time points at which rapid cognitive decline began. Moreover, different participants were identified for early or late decline subgroups across different cognitive domains, highlighting the heterogeneity of the process of cognitive decline. Importantly, the cognitive trajectory classes capture information about decline that is related to, but not the same as, the clinical diagnoses: despite the absence of a perfect alignment between the identified early or late decline groups and the diagnoses of dementia and MCI, higher incident dementia and incident MCI rates were observed when the number of domains in which an individual was clustered into the early decline group increased.

Understanding the long preclinical phase of AD is crucial for early intervention opportunities (Sperling et al., 2011). Linking heterogeneity in the extended preclinical phase with existing physical or biological markers of AD or related dementia may advance the knowledge of multiple pathways of cognitive aging. Our proposed flexible model, which results in the subgrouping of individuals based on their cognitive function trajectories, could provide a potential proxy measure for the onset of cognitive decline. Particularly, the early decline group identified through this model may function as a significant risk factor for preclinical AD or related dementia. Aiming to demonstrate the broader utility of these subgroupings, we also established connections with existing peripheral inflammatory biomarkers of cognitive aging. Our previous study observed cross-sectional correlations between higher levels of peripheral CD14, CD40L, and MPO; lower levels of peripheral sRAGE with lower scores in the WAIS-IV Similarities subtest, which was commonly used to measure cognitive performance in the executive function domain (Ho et al., 2018); and CD5L associated with brain volume biomarkers. In our current analysis, with the addition of longitudinal data and cognitive domain scores that harmonize information from separate tests, we have identified nominally significant additional associations: elevated levels of CD14 are associated with a higher risk of early cognitive decline in the executive function and CD40L are associated with higher risk for early decline in the memory domain, respectively.

Our study benefits from the large amount of longitudinal data the community-based FHS cohorts provide, and their comprehensive neuropsychological assessments together with the calibrated and harmonized domain global cognitive function scores. However, there are several limitations as well. First, the FHS participants are predominantly white. Therefore, our results may not be generalizable to wider populations. However, although accounting for a small proportion of study samples, our study includes participants from the multiethnic Omni 1 cohort. In addition, the modeling framework we chose, LCMM, typically suffers from computational convergence issues, especially when the number of model parameters to be estimated is large. Therefore, the determination of time points at which the rate of decline changes is pre-specified based on expert opinions and hence not identified among the data, which may affect the model’s generalizability as well. Moreover, while LCMM is designed to handle missing data under the assumption of missing at random, it does not explicitly account for dropout related to worsening cognitive function. However, the trajectories observed in our data show significant declines in cognitive scores over time, suggesting that this issue is not universal. Last, the modeling framework our study exploited is not prognostic but instead only exploratory. We investigated the heterogeneity in the longitudinal cognitive function profiles first, then summarized participants’ demographics and investigated the associations with existing biomarkers in a *post-hoc* manner. Therefore, in this *post-hoc* association analysis, the small sample size in the early decline groups, especially for the executive function domain, may lead to higher variability in effect size estimate and hence reduce the estimate stability and hinder the power of detecting significant association. Additionally, although we have selected the closest cognitive test time points included in the modeling of trajectory subgroups to the biomarker acquisition exam, the time differences between these two instances could be large and hence introduce additional variability. Due to the exploratory nature of our study, we did not apply multiple testing corrections to our association analyses with the five proteins, and reported nominally significant (*p* < 0.05) associations. Additional studies will be needed to confirm the reported associations and further validate our findings.

Recently, Li et al. (2022) proposed regularized finite mixture regression models to perform clustering for the identification of heterogeneity among the samples and enabled variable selections for the regression model components simultaneously, hence, the source of the heterogeneity can be identified. An interesting future direction of exploration could be to extend the regularized finite mixture regression model to incorporate both fixed effects and random effects for better handling longitudinal data and identifying sources of heterogeneity. This could also help build a prognostic model so that the groups characterized by unique cognitive decline patterns can be predicted using the selected corresponding features like participants demographics, *APOE* genotype, cardiovascular risk factors, and other existing AD or dementia biomarkers. Another future direction includes developing a framework for changepoint detection while clustering the time-course profiles in the meantime so that the computational burden from the traditional LCMM can be lifted and the change points can be identified from a data-driven approach.

In summary, our study offers a comprehensive exploration of cognitive decline heterogeneity among FHS participants, employing a flexible, robust, and reproducible model that clusters individuals based on longitudinal cognitive factor scores. Our study identifies class-related differential patterns in cognitive aging in the FHS, creates a proxy classification of cognitive decline subtypes among the FHS participants which are available for the wider scientific community to use in other projects. Overall, our study advances the understanding of cognitive decline heterogeneity and sets the stage for further investigations into early intervention strategies and personalized approaches to mitigate cognitive aging risks.

## Data Availability

Publicly available datasets from the Framingham Heart Study were analyzed in this study. This data can be found here: https://biolincc.nhlbi.nih.gov/studies/fhs/.
