# Supplementary material for: Modeling heterogeneity in cognitive trajectories in the Framingham Heart Study
Source: Front Aging Neurosci. 2025 Jun 25;17:1471154. doi: 10.3389/fnagi.2025.1471154 (PMC12238755; doi:10.3389/fnagi.2025.1471154)
Supplement: Supplementary file 1 [file Data_Sheet_1.pdf]

**Supplementary material for "Modeling heterogeneity in cognitive trajectories in the Framingham Heart Study"**

# 1 Mathematical details on the piecewise linear LCMM model

## 1.1 Piecewise linear mixed-effect model construction

For each subject $i$ in a sample of $N$ subject, if model the response variable $Y_i$ using simple piecewise linear regression with a point of time change T, and adjusting other time-invariant predictors $\mathbf{X}_i$, we will have

$$E(Y_i) = \beta_0 + \beta_1 t_i + \delta(t_i - T)_+ + \mathbf{X}_i^\top \boldsymbol{\gamma}$$

where

$$(t_i - T)_+ = (t_i - T)I(t_i - T) = \begin{cases} 0, & \text{if } t_i \leq T \\ t_i - T, & \text{if } t_i > T. \end{cases}$$

In this construction, $\beta_1$ represents the slope before the change time T and $\delta$ represents the change in slopes before and after the change time T.

Now consider each subject $i$ has $n_i$ repeated measures and $Y_{ij}$, for $j = 1, \ldots, n_j$ is the response measured at time $j$. Also, consider more than one but a set of time points $T_1 < T_2 < ... < T_K$, fixed for all subjects at which slopes of the trajectories may change. Then, the trajectories can be modeled via the following piecewise linear mixed-effect model (piecewise LMM):

$$Y_{ij} = \beta_0 + \beta_1 t_{ij} + \sum_{k=1}^{K} \delta_k (t_{ij} - T_k)_+ + \mathbf{X}_i^\top \boldsymbol{\gamma} + \mathbf{Z}_i(t_{ij})^\top \mathbf{u}_i + \epsilon_{ij}, \quad (1)$$

where $\delta_k$ is the change in slope before and after the change time $T_k$, $\forall k = 1, \ldots, K$. $\mathbf{X}_i$ is a vector consists of time-invariant covariates corresponding with the fixed effects $\boldsymbol{\gamma}$ and $\mathbf{Z}_i(t_{ij})$, which typically are functions of time $t_{ij}$, are associated with the random effects $\mathbf{u}_i$, which is

assumed to follow a zero-mean multivariate normal distribution with covariance matrix $\mathbf{D}$, i.e.,

$$\mathbf{u}_i \sim \mathrm{MVN}(\mathbf{0}, \mathbf{D});$$

and the residual $\epsilon_{ij}$ follows a normal distribution $\mathrm{N}(0, \sigma_\epsilon^2)$. Therefore we have the mean and variance:

$$\mathbb{E}(Y_{ij}) = \beta_0 + \beta_1 t_{ij} + \sum_{k=1}^{K} \delta_k (t_{ij} - T_k)_+ + \mathbf{X}_i^\top \boldsymbol{\gamma};$$

$$\mathrm{Var}(Y_{ij}) = \mathbf{Z}_i(t_{ij})^\top \mathbf{D} \mathbf{Z}_i(t_{ij}) + \sigma_\epsilon^2.$$

In the scenario we are considering, we also allow for random intercept and random slopes before and after each change point in the model. More specifically, we have $\mathbf{Z}_i(t_{ij})^\top = (1, t_{ij}, (t_{ij} - T_1)_+, \dots, (t_{ij} - T_K)_+)$, $\mathbf{u}_i^\top = (u_{i0}, u_{i1}, u_{i\delta_1}, \dots, u_{i\delta_K})$ where $u_{i\delta_k}$ is the change in random slope before and after $T_k$, $\forall k = 1, \dots, K$. Hence,

$$Y_{ij} = \beta_0 + \beta_1 t_{ij} + \sum_{k=1}^{K} \delta_k (t_{ij} - T_k)_+ + \mathbf{X}_i^\top \boldsymbol{\gamma} + u_{i0} + u_{i1} t_{ij} + \sum_{k=1}^{K} u_{i\delta_k} (t_{ij} - T_k)_+ + \epsilon_{ij}. \quad (2)$$

### 1.2 Piecewise linear LCMM model construction

Generalization from LMM to a latent class mixed-effect model (LCMM) is a relative straightforward step. We adopt the framework described in Proust-Lima, 2017 [1], which used a finite mixture of regression models to represent the latent class structure.

A $G-$component LCMM assumes the $N$ subjects come from a mixture of $G$ unobserved groups; each subject belongs to one and only one group (latent class) and each group is characterized by a distinct mean trajectory profile. T class membership variable, denoted as $c_i$ for $i, = 1, \dots, N$, is a discrete random variable and is typically unobserved. $c_i = g$ indicates that the $i^{th}$ subject is from the $g^{th}$ latent class for $g = 1, ..., G$. Then, by considering the piecewise LMM regression function described in Eq 1, for observation $i$ at time point $j$, the

overall regression be written as a weighted sum from $G$ group-specific regression functions:

$$f(Y_{ij}|\boldsymbol{\pi}_i, \boldsymbol{\Theta}_i) = \sum_{g=1}^{G} \pi_{ig} f_g(Y_{ij}|\Theta_{ig}, t_{ij}, \mathbf{X}, \mathbf{Z}_i(t_{ij})), \tag{3}$$

where $\boldsymbol{\pi}_i = (\pi_{i1}, ..., \pi_{iG})$, $\boldsymbol{\Theta}_i = (\Theta_{i1}, ..., \Theta_{iG})$ and $\Theta_{ig} = (\beta_{0g}, \beta_{0g}, \delta_{g1}, \ldots, \delta_{gK}, \boldsymbol{\gamma}_g, \mathbf{u}_{ig})$, and the mixing proportion $\pi_{ig}$ is the probability that $i^{th}$ subject belongs to the $g^{th}$ latent class, i.e., $\pi_{ig} = P(c_i = g)$, and

$$Y_{ij}|_{c_i=g} = \beta_{0g} + \beta_{1g} t_{ij} + \sum_{k=1}^{K} \delta_{gk}(t_{ij} - T_k)_+ + \mathbf{X}_i^\top \boldsymbol{\gamma}_g + \mathbf{Z}_i(t_{ij})^\top \mathbf{u}_{ig} + \epsilon_{ij}, \tag{4}$$

hence we constructed the piecewise linear LCMM.

Given that the finite mixture of regression framework is a clustering, i.e., an unsupervised learning framework, the true number of component $G$, is not observed and need to be estimated. Common practice in the clustering context is to fit the model with a range of all possible $G$s, e.g., for $G = 1, \ldots, 5$ and employ information criteria such as Bayesian Information Criteria (BIC [2]) to help select the model that fits the data best.

## 2 Piecewise linear LCMM model specification

The ideal case would be if we could pre-specify all potential change points and fit the "complete" piecwise linear LCMM described in Eq 3 and Eq 4. Then, a backward selection type of algorithm could be applied to select the change points that are significant. In the construction of the piecewise linear LCMM Eq 3, it is possible to specify either common or group-specific fixed effects, as well as variance-covariance matrices for the random effects. When specifying our model, we assumed group-specific fixed effects and random effect variance-covariance matrices to allow for flexibility. Notice that although in the "complete" model, all subjects starts with the same model by allowing for piecewise LMM at every prespecified time points of potential change, by allowing for group-specific fixed effect, subjects

identified in different groups may end up with different change points retained at the end of the backward selection.

In practice, potential change points can range from being as specific as the onset of clinical events, determined by clinicians, to as broad as occurring at regular intervals (e.g., every 5 years). However, it's widely recognized that the LMM and LCMM models can struggle to converge, especially in computer programs like R, when estimating a large number of parameters. Therefore, to alleviate the computation burden, we pre-specified two sets of change points, i.e., one is at age of $(65, 75, 85)$ in years, the other is at age of $(70, 80, 90)$ in years, and fit two complete models for all subjects separately. These two complete model will be fitted for all possible number of groups, which we specified as $G = 1, \ldots, 4$. For each of the 8 complete models as a starting point, backward selection algorithm are performed; resulting in 8 post-change-point-selection models with group-specified changing points selected for $G = 1, \ldots, 4$ groups. The final best model will be then evaluated by the best BIC among the 8 post-change-point-selection models.

We performed this procedure for all three cognitive function domains that we are interested in, i.e., memory, executive function, and language. The flexibility we brought in by the combination of group-specific fixed and random effects and backward selection will eventually allow for group specific change points selected for different cognitive domains.

## 3 Backward selection algorithm for the Piecwise linear LCMM

**Algorithm 1** Backward Selection Algorithm for the Piecewise Linear LCMM

**Input:** A specific number of classes $G$ (from $G = 1, \ldots, 4$) and one starting point set of change points $((65, 75, 85)$ or $(70, 80, 90))$
**Output:** A reduced model with significant change points and class predictors
**Step 0:** Fit the complete model and obtain model summary
**repeat**
    **Step 1:** Extract p-values for the change points
    **Step 2:** Remove the (change point, class) paired predictor with the highest p-value from the fixed effect and refit the model
    **if** for a change point, variables for **ALL** classes are removed **then**
        Exclude this change point from the random effect
    **end if**
    **Step 3:** Update the model formula and initial values accordingly, refit the model allowing for grid-search types of random start
**until** all variables remaining in the model have p-values $< 0.05$

## 4 Supplementary Tables

Supplementary Table 1. 10-fold cross validation performance in the memory domain.

| | | BIC select | Median posterior probability (IQR) | ARI with the full-data model predicted class | Median posterior probability (IQR) |
|---|---|---|---|---|---|
| | | Training data | | Testing data | |
| **Memory Domain** | iteration 1 | G = 3 | 0.78 (0.12) | 0.51 | 0.97 (0.08) |
| | iteration 2 | G = 3 | 0.74 (0.12) | 0.53 | 0.98 (0.07) |
| | iteration 3 | G = 4 | 0.79 (0.19) | 0.97 | 0.79 (0.13) |
| | iteration 4 | G = 4 | 0.79 (0.19) | 1.00 | 0.79 (0.23) |
| | iteration 5 | G = 4 | 0.79 (0.19) | 0.98 | 0.79 (0.19) |
| | iteration 6 | G = 4 | 0.79 (0.19) | 1.00 | 0.77 (0.17) |
| | iteration 7 | G = 3 | 0.90 (0.1) | 0.61 | 0.98 (0.08) |
| | iteration 8 | G = 4 | 0.79 (0.19) | 0.98 | 0.78 (0.21) |
| | iteration 9 | G = 4 | 0.74 (0.12) | 0.43 | 0.74 (0.13) |
| | iteration 10 | G = 3 | 0.91 (0.1) | 0.66 | 0.95 (0.09) |

Supplimentary Table 2. 10-fold cross validation performance in the executive function domain.

| | | BIC select | Median posterior probability (IQR) | ARI with the full-data model predicted class | Median posterior probability (IQR) |
|---|---|---|---|---|---|
| | | Training data | | Testing data | |
| **Executive Function Domain** | iteration 1 | G = 2 | 1.00 (0.02) | 1.00 | 1.00 (0.01) |
| | iteration 2 | G = 2 | 1.00 (0.02) | 0.92 | 0.99 (0.02) |
| | iteration 3 | G = 2 | 1.00 (0.02) | 0.79 | 1.00 (0.02) |
| | iteration 4 | G = 1 not converge | | | |
| | iteration 5 | G = 2 | 1.00 (0.02) | 1.00 | 1.00 (0.02) |
| | iteration 6 | G =2 | 1.00 (0.02) | 1.00 | 1.00 (0.02) |
| | iteration 7 | G =2 | 1.00 (0.01) | 1.00 | 1.00 (0.01) |
| | iteration 8 | G = 2 | 1.00 (0.01) | 1.00 | 1.00 (0.01) |
| | iteration 9 | G = 2 | 1.00 (0.01) | 1.00 | 1.00 (0.01) |
| | iteration 10 | G =2 | 1.00 (0.02) | 0.94 | 0.99(0.02) |

Supplementary Table 3. 10-fold cross validation performance in the language domain.

| | | **BIC select** | **Median posterior probability (IQR)** | **ARI with the full-data model predicted class** | **Median posterior probability (IQR)** |
|---|---|---|---|---|---|
| | | Training data | | Testing data | |
| **Language Domain** | iteration 1 | G = 3 | 0.90 (0.13) | 0.91 | 0.91 (0.12) |
| | iteration 2 | G = 3 | 0.89 (0.14) | 0.86 | 0.90 (0.13) |
| | iteration 3 | G = 3 | 0.70 (0.22) | 0.30 | 0.71 (0.20) |
| | iteration 4 | G = 3 | 0.89 (0.14) | 0.96 | 0.89 (0.13) |
| | iteration 5 | G = 3 | 0.89 (0.14) | 0.94 | 0.90 (0.14) |
| | iteration 6 | G = 3 | 0.89 (0.14) | 0.96 | 0.89 (0.13) |
| | iteration 7 | G = 3 | 0.89 (0.14) | 0.95 | 0.88 (0.14) |
| | iteration 8 | G = 3 | 0.70 (0.22) | 0.27 | 0.70 (0.22) |
| | iteration 9 | G = 3 | 0.89 (0.14) | 0.95 | 0.88 (0.14) |
| | iteration 10 | G = 3 | 0.90 (0.14) | 0.96 | 0.88 (0.16) |

Supplementary Table 4. Association of the protein biomarkers with the piecewise linear LCMM identified subclasses as early decline vs. steady/late decline in language domain.

| **Biomarker** | **Odds Ratio** | **Standard Error** | ***p*-value** |
|---|---|---|---|
| Main analysis sample (n=1617) | | | |
| CD14 | 0.96 | 0.21 | 0.86 |
| CD5L | 1.01 | 0.15 | 0.94 |
| CD40L | 0.84 | 0.18 | 0.32 |
| sRAGE | 1.08 | 0.13 | 0.53 |
| MPO | 1.22 | 0.13 | 0.14 |
| Sensitivity analysis sample (n=907) | | | |
| CD14 | 0.92 | 0.27 | 0.78 |
| CD5L | 0.81 | 0.16 | 0.19 |
| CD40L | 0.70 | 0.25 | 0.15 |
| sRAGE | 0.81 | 0.25 | 0.40 |
| MPO | 1.02 | 0.15 | 0.91 |

## References

[1] Cécile Proust-Lima, Viviane Philipps, and Benoit Liquet. Estimation of extended mixed models using latent classes and latent processes: The r package lcmm. *Journal of Statistical Software*, 78(i02), 2017.

[2] Gideon Schwarz. Estimating the dimension of a model. *The annals of statistics*, pages 461–464, 1978.
